# Development of a Prognostic Score Using the Complete Blood Cell Count for Survival Prediction in Unselected Critically Ill Patients

**DOI:** 10.1155/2013/105319

**Published:** 2013-02-28

**Authors:** Fang Chongliang, Li Yuzhong, Shi Qian, Liu Xiliang, Liu Hui

**Affiliations:** ^1^Second Affiliated Hospital of Dalian Medical University, Dalian 116023, China; ^2^College of Medical Laboratory, Dalian Medical University, Dalian 116044, China; ^3^Center Hospital of Dalian, Dalian 116033, China

## Abstract

*Objective*. The purpose of this study was to develop a new prognostic scoring system for critically ill patients using the simple complete blood cell count (CBC). *Methods*. CBC measurements in samples from 306 patients in an intensive care unit were conducted with automated analyzers, including levels of neutrophils, lymphocytes, erythrocytes, hemoglobin, and platelets. The time of sampling and the time of death were recorded. *Z* values were calculated according to the measured values, reference mean values, and standard deviations. The prognostic score was equivalent to the median of the *Z* value of each of the measured parameters. *Results*. There was a significant correlation between survival time and neutrophil, lymphocyte, and platelet levels (*P* < 0.05). Prognostic scores were calculated from the *Z* value of these three parameters. Survival times decreased as the prognostic score increased. *Conclusions*. This study suggests that a model that uses levels of neutrophils, lymphocytes, and platelets is potentially useful in the objective evaluation of survival time or disease severity in unselected critically ill patients.

## 1. Introduction

Although accurate prediction of survival is essential for palliative care, the numerous clinical tools currently available that attempt to determine how long a patient is likely to live remain inappropriate [1, 2]. The clinical significance of studies on survival predictors in critically ill patients is hindered by the difficulty of finding objective and quantitative common predictors of death. On the basis of such studies, performance status, including parameters such as anorexia, weight loss, dysphagia, dyspnea, and cognitive failure, has been found to correlate most strongly with survival [3–5]. However, the nature of this subjective and qualitative data tends to preclude precise specification. Some prognostic scoring systems with performance status, clinical symptoms, and biochemical parameters help to guide accurate prediction, such as the acute physiology and chronic health evaluation (APACHE), yet are considered too complex for general clinical use [6]. Therefore, the prediction of clinical events with laboratory parameters, including complete blood cell count (CBC), has become an increased focus of research. Accumulating evidence indicates that the CBC is an effective predictor of mortality in many disease states, including hematological disease [7–9], neoplasms [10–12], and diseases of the circulatory system [13–15].

Biomarkers in fluids offer the opportunity for more objective and reproducible measurement. They are not only able to provide a reliable and objective basis for screening and for diagnosis, but they can also measure disease severity [16–18]. The CBC demonstrates nonspecific changes in a variety of critical illnesses [19–21]. Therefore, nonspecific changes in the CBC in critically ill patients could be considered a key prognostic factor in the evaluation of survival prediction in these patients [7–15]. Accordingly, it is possible that the CBC could be used as a predictor of survival in unselected critically ill patients. The current study performed a quantitative analysis on changes in the CBC in critically ill patients. Furthermore, we developed a new prognostic scoring system to evaluate survival times with sensitive parameters specifically for critically ill patients.

## 2. Subjects and Methods

### 2.1. Subjects

Critically ill patients recruited into this study received treatment in an intensive care unit (ICU) between July 2006 and December 2010 at three hospitals (Second Affiliated Hospital of Dalian Medical University, Center Hospital of Dalian, and Third Hospital of Dalian, China). Inclusion criteria were availability of data on CBCs in medical records and death within one year of CBC measurement. Exclusion criteria were primary diseases from systemic hematological disorders and accidental injuries. A total of 306 subjects were selected (170 males, 136 females; mean age 65.3 ± 15.5 years). The main diseases were cardiovascular and cerebrovascular (*n* = 69), chronic obstructive pulmonary disease (*n* = 13), various types of tumors (*n* = 86), and other diseases (*n* = 138). 

The time of the observation, that is, the CBC measurement, and the time of death were recorded. The date of death was obtained from medical records and residence queries. On the basis of survival time, subjects were divided into 8 groups (Day 1, Day 2, Day 3, Day 4, Day 5, Days 6–30, Days 31–180, and Days 180–365). The study was conducted in accordance with the Declaration of Helsinki, and the study was approved by the Institutional Ethics Committee of Dalian Medical University.

### 2.2. CBC Data

CBC data was obtained from medical records. CBC measurement, which included levels of neutrophils (Neu, 3.5 ± 1.2 · 10^9^/L), lymphocytes (Lym, 2.2 ± 0.6 · 10^9^/L), red blood cells (RBC, 4.5 ± 0.6 · 10^12^/L), hemoglobin (HB, 142 ± 20 g/L), and platelets (Plt, 210 ± 53 · 10^9^/L), was performed with an automatic analyzer, using standard commercial reagent kits. All the analyzers met the requirements of external quality assessment in Dalian, China.

### 2.3. Statistical Analysis and Establishment of the Model

Correlation coefficient (Spearman's method) was used to assess the correlation between survival time and biochemical parameters (alpha = 0.05, two-tailed test). The significant indicators were used to set up the prognostic score. On the basis of the reference range, we obtained mean values ($\bar{X}$) and standard deviation (*S*). The *Z* values were calculated according to the following formulas, where *X* is the measured value:

 Neu, Lym: $Z=(X-\bar{X})/S$.
 RBC, HB, Plt: $Z=(\bar{X}-X)/S$.

The prognostic score was equivalent to the median of the *Z* value of each of the measured parameters. For *Z* values of Neu, Lym, and Plt of 1.36, 1.70, and 1.45, respectively, for example, the prognostic score was 1.45.

## 3. Results

The original data for survival time in this cohort of critically ill patients and levels of Neu, Lym, RBC, HB, and Plt is shown in Table 1. The relationship between survival time and CBC levels as determined by correlation analysis is shown in Table 2. The Neu, Lym, and Plt levels are considered predictors. Prognostic scores were calculated from the *Z* value of Neu, Lym, and Plt levels, as shown in Table 3. Survival time decreased as the prognostic scores increased.

## 4. Discussion

Natural death for the majority of people can be considered a process of loss of organ function. Compensatory or stress responses will occur during this process. The CBC contributes to the data used to assess these reactions and potentially offers the opportunity for a more objective measurement [7–15]. Therefore, identification of the CBC in unselected critically ill patients is essential in our understanding of the impairment and restorative processes that occur during the end of life. In the current study, Neu and Lym levels were negatively correlated with survival time, while Plt level was positively correlated, indicating that these three parameters could have potential in the prediction of survival in critically ill patients.

Theoretically, the three parameters change independently. Abnormal changes in two or three of them can be considered as nonspecific systemic changes that are associated with death, and abnormal changes in a single indicator may be specific. There may be no correlation between a change in a single indicator and the occurrence of death. Thus, we transformed measurement data into *Z* values in order to assess the comparability of change in measured values. The median of the *Z* value of each of the measured parameters (prognostic score) can be considered a quantitative evaluation of holistic changes rather than a change in only one indicator. 

As data transformation into *Z* values was conducted according to the reference range of each item, theoretically the score is equivalent to zero, and the higher the observed score, the greater the risk of death. Our results show that survival time decreases as the prognostic score increases, suggesting that a model using Neu, Lym, and Plt levels is potentially useful in the objective evaluation of survival time in critically ill patients.

Multiple analysis is a basic tool for developing a risk model [22–24]. Large populations of several thousand individuals are required to perform the linear multiple regression. Our mathematical model was established on the basis of normal reference ranges rather than multiple analyses. The patients could be considered as a separate validation population. Compared to traditional methods, our method for establishing a mathematical model had a higher efficiency.

We believe that disease severity when patients are admitted to ICU is not easy to determine objectively. Therefore, time of death is a reliable indicator of disease severity. The subjects needed to be standardized, thus rendering the results comparable; thus, subjects were divided on the basis of survival time. Our results showed that the predictive score in the group that survived 181–365 days was close to zero, close to the theoretically normal score. This implied that the score would be zero or close to zero in patients who survive more than 365 days, but this group was not observed in this study. 

The hypothesis for this study was that CBC results are associated with imminent death and are unlikely to be related to death occurring in the longer term, as a result of many confounding variables. The current study demonstrates a responsiveness of the CBC to death, within a period of approximately one week, which is consistent with our hypothesis. CBC is a conventional laboratory test and contains much information. Our results may be useful for the development of a validated scoring system for survival prediction or disease severity. Further prospective randomized controlled trial studies are warranted to validate the usefulness of these determinants for accurate survival predictions.

## Figures and Tables

**Table 1:** Survival time and levels of baseline CBC indicators in critically ill patients.

Group	Survival median day	*N*	Blood cell count (mean ± SD)				
				
			Neu	Lym	RBC	HB	Plt
Day 1	1	59	14.0 ± 11.3	2.1 ± 1.9	3.5 ± 1.2	108.1 ± 34.2	140.5 ± 101.8
Day 2	2	64	12.3 ± 6.5	1.4 ± 1.0	3.6 ± 1.2	111.1 ± 38.3	150.3 ± 102.0
Day 3	3	29	9.5 ± 5.6	0.9 ± 0.6	3.7 ± 1.1	111.5 ± 34.8	138.1 ± 75.2
Day 4	4	21	10.6 ± 7.3	1.5 ± 1.3	3.5 ± 1.0	107.3 ± 30.5	152.4 ± 94.1
Day 5	5	34	10.9 ± 7.1	1.2 ± 0.7	3.6 ± 0.8	107.3 ± 26.1	167.0 ± 81.0
Days 6–30	14	38	10.1 ± 5.4	1.2 ± 0.7	3.4 ± 0.7	106.5 ± 22.1	171.8 ± 100.1
Days 31–180	91	37	6.9 ± 4.5	1.4 ± 0.7	3.7 ± 0.8	114.4 ± 22.2	209.9 ± 102.2
Days 181–365	296	24	5.2 ± 3.0	1.6 ± 0.7	4.0 ± 0.8	121.8 ± 28.7	223.3 ± 92.8

**Table 2:** Relationship between survival time and levels of CBC indicators.

Correlation		Neu	Lym	RBC	HB	Plt
Survival time	*r*	−0.142	−0.168	0.008	−0.015	0.125
	*p*	0.012	0.003	0.892	0.788	0.027

**Table 3:** Distribution of prognostic scores in different survival time groups.

Group	Score (Quartile)		
	25th	50th	75th
Day 1	0.41	2.12	2.87
Day 2	0.18	1.63	2.62
Day 3	−0.47	1.19	2.25
Day 4	−0.21	0.73	2.35
Day 5	−0.12	0.73	1.62
Days 6–30	−0.08	0.82	1.76
Days 31–180	−0.98	−0.06	0.50
Days 181–365	−0.96	−0.34	0.33
